# Do NSAIDs and ASA Cause More Upper Gastrointestinal Bleeding in Elderly than Adults?

**DOI:** 10.1155/2016/8419304

**Published:** 2016-01-05

**Authors:** Hakan Kocoglu, Basak Oguz, Hakan Dogan, Yildiz Okuturlar, Mehmet Hursitoglu, Ozlem Harmankaya, Yuksel Altuntas, Abdulbaki Kumbasar

**Affiliations:** ^1^Department of Internal Medicine, Bakirkoy Dr. Sadi Konuk Education and Research Hospital, Istanbul, Turkey; ^2^Department of Internal Medicine, Istanbul Bilim University Medical Faculty, Florence Nightingale Hospital, Istanbul, Turkey; ^3^Department of Internal Medicine, Izmir Bozyaka Education and Research Hospital, Izmir, Turkey; ^4^Department of Endocrinology and Metabolism, Sisli Hamidiye Etfal Education and Research Hospital, Istanbul, Turkey

## Abstract

*Purpose*. NSAIDs and ASA may cause upper gastrointestinal bleeding (UGIB) both in adults and in elderly. There is no study that compares this increased bleeding risk between adult and elderly subjects.* Methods*. A total of 524 patients with UGIB were included in this study. The data of patients were, respectively, analyzed.* Results*. NSAIDs and ASA-associated UGIB rates were similar between <65 years (345 patients) (group 1) and ≥65 years (179 patients) (group 2) (28.4% versus 23.5%, *p* = 0.225 and 13% versus 19%, *p* = 0.071, resp.). Warfarin-associated UGIB was found significantly higher in group 2 than group 1. Elderly patients with NSAID-associated UGIB had significantly higher length of stay (LoS) and CoH than adult patients with NSAID-associated UGIB (*p* = 0.002 and 0.001, resp.). Elderly patients with ASA-associated UGIB had significantly higher CoH than adult patients with NSAID-associated UGIB.* Conclusions*. Using NSAIDs without gastroprotective drugs or using ASA with gastroprotective drugs in elderly patients is as safe as in adult patients. Not only should adding gastroprotective drugs to ASA or NSAID be based on their risk of UGIB, but the cost of hospitalization of ASA or NSAID-associated UGIB should be considered.

## 1. Introduction

The older population is growing faster than the total population in all regions of the world and more than 1% of elderly people are hospitalized each year because of upper gastrointestinal bleeding (UGIB) [1, 2]. Advanced age has been consistently identified as a risk factor for mortality among patients with UGIB, presumably because of a higher prevalence of comorbid illnesses in the elderly [3, 4]. The incidence and outcome of gastrointestinal bleeding in elderly people can also be influenced by the use of aspirin, other nonsteroidal anti-inflammatory drugs, and other antiplatelet and anticoagulant agents [5, 6].

Previous studies have shown that almost 30% of the elderly people have been taking NSAIDs (including ASA) [7, 8]. NSAIDs and ASA have widespread usage in various diseases and their relationship with UGIB is well known. In all ages, including elderly, people using NSAIDs or ASA tended to have higher UGIB risk than those who do not use [9–12].

Several studies showed that NSAIDs and ASA are risk factors for UGIB both in adults and in elderly patients, but whether NSAIDs and ASA cause more UGIB events in elderly than adults, or vice versa, is debatable. So, in this study, we have tried to answer the question of “do NSAIDs and ASA cause more UGIB events in elderly than adults?”

## 2. Materials and Methods

A total of 524 patients' data who consecutively admitted to our hospital with the diagnosis of UGIB over the last three years were obtained from the hospital records and from patients or their relatives as well. The diagnosis of UGIB in all patients was proven by endoscopy, physical examination (e.g., melena), and/or laboratory values (decrease in hematocrit value that is not caused by other reasons). The data of patients, including age, gender, mortality, the number of comorbid diseases, length of hospital stay (LoS), cost of hospitalization (CoH), number of transfusion of packed erythrocytes, and the use of ASA and/or NSAIDs, were, respectively, analyzed.

*Statistical Analysis*. Obtained data were analyzed by using SPSS 22 software (IBM Corp., Armonk, NY). During the evaluation of study variables, categorical and continuous variables were summarized using the descriptive statistics (e.g., median, range, frequency, and percentage) and compared with Chi-square, Kruskal Wallis, and Mann-Whitney *U* tests, as appropriate. A value of *p* < 0.05 was considered as statistically significant.

## 3. Results

The hospital-based registry included a total of 524 patients that consisted of 345 patients younger than 65 years (group 1) and 179 patients aged 65 years or older (group 2). There was a statistical significance in mean age between two groups (77.59 ± 7.70 versus 42.83 ± 13.9 years, resp., *p* = 0.0001). Female and male percentages of group 1 and group 2 were 22.9%, 77.1% and 46.4%, 53.6%, respectively. Mean duration of hospital stay was 6.35 ± 4.94 days in all groups. Mean duration of hospital stay was statistically significant between group 1 and group 2 (5.59 ± 3.89 versus 7.8 ± 6.23 days, resp., *p* = 0.0001). The number of patients who had comorbid disease was significantly higher in group 2 than group 1 (87.2% versus 40.7%, resp., *p* = 0.0001). Also number of transfusion of packed erythrocytes and number of comorbid diseases were significantly higher in group 2 than group 1 (2.72 ± 2.30 versus 1.91 ± 2.17 unit and 1.83 ± 1.30 versus 0.65 ± 0.95, resp., *p* = 0.0001 for both) (Table 1).

The most common causes of bleeding in elderly patients were bulbar (duodenal) ulcer (28.4%), gastric malignant ulcer/mass (10.8%), and erythematous pangastritis (10.2%). And the most common causes of bleeding in patients younger than 65 years were bulbar (duodenal) ulcer (50.5%), gastric ulcer (9.8%), and erythematous pangastritis (8.6%) (the data was not shown).

Group 2 had higher mortality rates than group 1 (10.1% versus 2%, resp., *p* = 0.0001). There were no differences in use of NSAIDs and ASA between group 1 and group 2 (28.4% versus 23.5%, *p* = 0.225, and 19% versus 13%, *p* = 0.071, resp.). None of the NSAIDs-user patient was taking gastroprotective medicines (e.g., proton-pump inhibitors). In all ASA-user patients, 3 of 45 (4.4%) patients in group 1 and 1 of 34 (2.94%) patients in group 2 were not taking gastroprotective medicines (*p* > 0.05). There were no differences in ASA-associated UGIB-related mortality and NSAIDs-associated UGIB-related mortality rates between group 1 and group 2 (*p* was 0.250 and 0.524, resp.). Also warfarin-associated UGIB was found significantly higher in group 2 than group 1 (*p* = 0.0001) (Table 2).

The mean CoH for patients with UGIB was $413.98 ± 374.5 (US Dollar). Also there was a positive correlation between patients' age and CoH (*r* = 0.293; *p* = 0.001). We also compared the LoS (day) and CoH (in US Dollar [exchange currency; 2 Turkish Liras = 1 US Dollar]) according to whether taking warfarin, ASA, or NSAID and patients' age (Table 3). LoS and CoH were significantly higher in patients (both adult and elderly) with warfarin than those without warfarin (*p* was 0.002 and 0.001, resp.). When we compared patients with and without ASA, only CoH, but not LoS, was significantly different (*p* = 0.024). Elderly patients with ASA had significantly higher CoH than adult patients with ASA and all patients without ASA (*p* was 0.007 and 0.013, resp.). Adult patients with NSAID had significantly lower LoS and CoH than elderly patients with NSAID and all patients without NSAID (see Table 3 for *p* values).

## 4. Discussion

In our study, female and male percentages of patients younger than 65 years and patients aged 65 years or older were found similar with previous studies [13, 14]. Viviane et al. determined the mean LoS for UGIB as 4.4 ± 2.7 days. In our study, it was 6.35 ± 4.94 days. Also mean LoS of patients younger than 65 years (7.8 ± 6.23 days) was found significantly higher than patients younger than 65 years (5.59 ± 3.89 days). As we know that having comorbid illness increases the LoS [15, 16], the difference between elderly and adult patients' hospital stay duration could be explained by having more comorbidities in elderly patients.

Previous studies have shown that peptic ulcer (includes gastric ulcer, duodenal ulcer, and peptic ulcer not otherwise specified) is the most common cause of bleeding in elderly patients [6]. In our study, the most common cause of bleeding was bulbar (duodenal) ulcer in both groups; but the second common cause was gastric malignant ulcer/mass in the elderly group whereas gastric ulcer was the second cause in adult patients.

According to our study, warfarin-user elderly patients had more UGIB than adult patients. In a study by Chen et al. which is consistent with our finding, age >65 years old has been found an independent risk factor for warfarin-associated gastrointestinal bleeding [17]. Pilotto et al. have stated in their study that elderly users of acute NSAIDs and regular-dose ASA have increased risk of UGIB [10], but in their study they did not compare these risks with adult subjects. We showed that UGIB risk of NSAIDs (if not cotreated with gastroprotective drugs) is not different in both groups (adult patients and elderly). Similarly UGIB risk of ASA (if cotreated with gastroprotective drugs) is not different in both groups (adult patients and elderly). In this study, we have also determined that there were no differences in ASA-associated UGIB-related mortality and NSAIDs-associated UGIB-related mortality rates between two groups. As we do not have enough number of patients who were treated with ASA but not with gastroprotective drugs, we cannot state any particular comment on this situation.

The mean CoH for patients with UGIB was $413.98 ± 374.5. Also there was a positive correlation between patients' age and CoH. Cryer et al. [18] have investigated the economics of UGIB in US and they have determined the mean CoH for patients with UGIB as $13,059. Also 12-month cost of a patient with UGIB, including total healthcare, medical, and pharmacy costs, was determined as $20,405. And they have shown that patients with UGIB experienced significantly higher 12-month health-resource utilization and costs than patients without UGIB. The other important point is that according to Cryer et al.'s study, patients who experienced UGIB had significantly higher cost of UGI-related filled prescription than general population ($558 versus 141). Thus cost-effectiveness studies are needed to examine the long-term economic benefits obtained from managing and preventing UGIB with medication.

According to our study, patients with warfarin-associated UGIB had higher CoH than all patients with UGIB without warfarin. Also, elderly patients with ASA-associated UGIB had higher CoH than adult patients with ASA-associated UGIB and all patients with UGIB without ASA. Interestingly, adult patients with NSAID-associated UGIB had lower LoS and CoH than elderly patients with NSAID-associated UGIB and all patients with UGIB without NSAID.

## 5. Conclusion

A positive correlation was determined between patients' age and cost of hospitalization.

Even though elderly patients are more prone to have warfarin-associated UGIB, physicians should be more careful when prescribing warfarin in not only elderly patients but also adult patients as those patients with warfarin-associated UGIB had higher CoH than patients with UGIB without warfarin.

When we consider only UGIB risk, using NSAIDs without gastroprotective drugs in elderly patients is as safe as in adult patients. But, even though there was no difference in the risk of NSAID-associated UGIB in between elderly and adult patients without gastroprotective drugs, elderly patients with NSAID-associated UGIB had significantly higher LoS and CoH than adult patients with NSAID-associated UGIB.

Similarly when ASA is prescribed with gastroprotective drugs in elderly, it can be used in elderly patients as safely as in adult patients. But, even though there was no difference in the risk of ASA-associated UGIB in between elderly and adult patients with gastroprotective drugs, elderly patients with ASA-associated UGIB had significantly higher CoH than adult patients with NSAID-associated UGIB.

Not only should adding gastroprotective drugs to ASA or NSAID be based on their risk of UGIB, but the cost of hospitalization of their-associated UGIB should be considered.

## Figures and Tables

**Table 1:** Comparison of patients <65 years and patients ≥65 years in regard to age, length of stay, number of transfusion of packed erythrocytes, and number of comorbid illness.

	Patients <65 years (*n* = 345)			Patients ≥65 years (*n* = 179)			*p*
	Mean ± SD	Minimum	Maximum	Mean ± SD	Minimum	Maximum	
Age (years)	42.83 ± 13.9	16	64	77.59 ± 7.70	65	97	**0.0001**
Length of stay (days)	5.59 ± 3.89	1	33	7.8 ± 6.23	1	39	**0.0001**
Number of transfusion of packed erythrocytes (unit)	1.91 ± 2.17	0	12	2.72 ± 2.30	0	15	**0.0001**
Number of comorbid illness	0.65 ± 0.95	0	4	1.83 ± 1.30	0	6	**0.0001**

**Table 2:** Comparison between patients <65 years and patients ≥65 years in regard to gender, clinical outcome, comorbid illness, ASA and NSAID use, and mortality.

	Patients <65 years (*n* = 345)		Patients ≥65 years (*n* = 179)	
	*n*	%	*n*	%
Gender				
Male	266	77.1	96	53.6
Female	79	22.9	83	46.4
Clinical outcome				
Dead	7	2	18	10.1
Discharged	338	98	161	89.9
*p*	**0.0001**			
Comorbid illness				
Yes	204	40.7	23	87.2
No	140	59.3	156	12.8
*p*	**0.0001**			
Warfarin use				
Yes	15	4.3	29	16.2
No	330	95.7	150	83.8
*p*	**0.0001**			
ASA use				
Yes	45	13	34	19
No	300	87	145	81
*p*	0.071			
NSAID use				
Yes	98	28.4	42	23.5
No	247	71.6	137	76.5
*p*	0.225			
Mortality rates of ASA-users				
Not ASA-users	7	100	15	83.3
ASA-users	0	0	3	16.7
*p*	0.250			
Mortality rates of NSAIDs-users				
Not NSAID-users	7	100	17	94.4
NSAID-users	0	0	1	5.6
*p*	0.524			

**Table 3:** Comparison of the length of stay and cost of hospitalization according to whether taking warfarin, ASA, or NSAID and patients' age.

	Group 1	Group 2	Group 3	*p* ^a^	*p* ^b^		
	Adult warfarin+	Elderly warfarin+	Warfarin−		1 vs 3	2 vs 3	1 vs 2
	*n* = 15	*n* = 29	*n* = 475				
	Mean ± SD (median)	Mean ± SD (median)	Mean ± SD (median)				
Length of stay (day)	8.53 ± 7.73 (6)	9.97 ± 7.36 (9)	6.11 ± 4.53 (5)	0.002^*∗∗*^	0.219	0.001^*∗∗*^	0.297
Cost of hospitalization (US Dollar)	554.88 ± 412.62 (543.2)	618.47 ± 421.39 (574.25)	397.59 ± 366.11 (320.65)	0.001^*∗∗*^	0.047^*∗*^	0.001^*∗∗*^	0.393

	Adult ASA+	Elderly ASA+	ASA−	*p* ^a^	1 vs 3	2 vs 3	1 vs 2
	*n* = 43	*n* = 35	*n* = 441				
	Mean ± SD (median)	Mean ± SD (median)	Mean ± SD (median)				

Length of stay (day)	6.09 ± 3.32 (5)	7.89 ± 6.99 (6)	6.31 ± 4.85 (5)	0.362	0.519	0.193	0.427
Cost of hospitalization (US Dollar)	349.72 ± 256.12 (303.25)	514.29 ± 309.21 (455.10)	412.87 ± 387.27 (337.65)	0.024^*∗*^	0.408	0.013^*∗*^	0.007^*∗∗*^

	Adult NSAID+	Elderly NSAID+	NSAID−	*p* ^a^	1 vs 3	2 vs 3	1 vs 2
	*n* = 98	*n* = 42	*n* = 379				
	Mean ± SD (median)	Mean ± SD (median)	Mean ± SD (median)				

Length of stay (day)	5.05 ± 2.58 (5)	7.24 ± 4.29 (6)	6.65 ± 5.38 (5)	0.007^*∗∗*^	0.012^*∗*^	0.134	0.002^*∗∗*^
Cost of hospitalization (US Dollar)	331.88 ± 301.26 (265.85)	494.98 ± 402.44 (427.75)	426.92 ± 385.18 (347.35)	0.005^*∗∗*^	0.013^*∗*^	0.111	0.001^*∗∗*^

^a^Kruskal-Wallis test, ^b^Mann-Whitney *U* test, ^*∗*^ *p* < 0.05, and ^*∗∗*^ *p* < 0.01.
